# Fasciculation intensity and limb dominance in amyotrophic lateral sclerosis: a muscle ultrasonographic study

**DOI:** 10.1186/s12883-022-02617-1

**Published:** 2022-03-11

**Authors:** Yo-ichi Suzuki, Kazumoto Shibuya, Sonoko Misawa, Tomoki Suichi, Atsuko Tsuneyama, Yuta Kojima, Keigo Nakamura, Hiroki Kano, Mario Prado, Satoshi Kuwabara

**Affiliations:** Department of Neurology, Graduate School of Medicine, Chiba University, 1-8-1 Inohana, Chuo-ku, Chiba, 260-8670 Japan

**Keywords:** Ultrasonography, Amyotrophic lateral sclerosis, Fasciculation, Handedness, Exercise

## Abstract

**Background and purpose:**

Muscle ultrasonography has been increasingly recognized as a useful tool for detection of fasciculations. Separately, concordance between dominant hand and onset side has been reported in amyotrophic lateral sclerosis (ALS). The aim of this study was to reveal the distribution of fasciculations in the whole body, focusing on handedness.

**Methods:**

In 106 consecutive patients with ALS, muscle ultrasonography was systematically performed in 11 muscles (the tongue, and bilateral biceps brachii, 1st dorsal interosseous [FDI], T10-paraspinalis, vastus lateralis and tibialis anterior muscles). The fasciculation intensity was scored from 0 to 3 for each muscle.

**Results:**

Fasciculations were more frequently found in the limb muscles than the tongue and paraspinalis. Side and handedness analyses revealed that fasciculation intensity in FDI was significantly more prominent on the right (median [inter-quartile range] 2 [0 - 3]) than left (1.5 [0 - 3]; *p* = 0.016), and in the dominant hand (2 [1 - 3]) than non-dominant side (1.5 [0 - 3]; *p* = 0.025). The differences were greater in patients with upper limb onset. There were no side differences in the lower limb muscles. Multivariate analyses showed that male patients had more frequent fasciculations in the dominant FDI (β = 0.22, *p* < 0.05).

**Conclusion:**

More intensive fasciculations are present in the FDI in the dominant hand and gender might be associated with fasciculation intensities. This distribution pattern of fasciculations might be associated with pathogenesis of ALS.

## Introduction

Amyotrophic lateral sclerosis (ALS) is a devastating disease, characterized by upper and lower motor neuron degeneration. For ALS diagnosis, the revised El Escorial diagnostic criteria were proposed [1]. In the criteria, upper and lower motor neuron signs must be found. While the specificity of this criteria is substantially high, the sensitivity is not so satisfactory [2]. As such, to increase the sensitivity of ALS diagnosis, Awaji and latest Gold Coast diagnostic criteria were advocated [3, 4]. Wide-spread and prominent fasciculations are characteristic features in ALS [5]. Therefore, in Awaji and latest Gold Coast criteria, fasciculations are regarded as an equivalent finding of acute denervation, and several studies have shown that Awaji and latest Gold Coast criteria increased the diagnostic sensitivity, compared with revised the El Escorial criteria [6–8].

Recently, the usefulness of ultrasonography for neuromuscular disorders has been emphasized [9]. Previous studies identified that ultrasonography is a useful tool to detect fasciculations in ALS, and increases the diagnostic sensitivity of ALS [10]. While conventional needle electromyography (EMG) can record the electrical activity from a hemisphere of radius of about 1 mm, the pick-up areas of ultrasonography are much larger than that, because the echo probe is bigger than that [11, 12]. Ultrasonography is assumed to find fasciculations widely within an individual muscles and increase diagnostic sensitivities [12].

Separately, ALS is characterized with several characteristic patterns of weakness. Distal muscles are frequently affected, compared to proximal muscles [13]. Moreover, concordance between site of onset and limb dominance has been reported [14]. Additionally, preferential wasting of the hand muscles in the thenar side and relative sparing of hypothenar muscles have been reported in ALS and called as split hand [15, 16]. Therefore, ALS disease process would differently affect each muscle in the body.

The aim of the present study was to reveal fasciculation distributions and patterns in ALS, focusing on the limb dominance or right-left differences. Findings of the present study may be useful not only for the diagnosis but also for investigating the underlying pathophysiology of motor neuron death in ALS.

## Methods

### Subjects

A total of 106 consecutive patients with ALS, who were seen at the Chiba University Hospital between February 2014 and November 2017, and diagnosed with possible, probable or definite ALS according to the revised El Escorial criteria, were included into this study [1]. Their clinical data, including gender, age at examinations, body mass index, disease duration, site of onset and handedness, were retrospectively reviewed. Ultrasonography was performed by well-trained examiners (over 5year experience) before the time of diagnosis [10]. Examiners were blinded to patient’s dominant hand. All subjects gave written informed consent to the procedures. This study was approved by the Ethics Committee of Chiba University School of Medicine (#1897). All methods were performed in accordance with the relevant guidelines and regulations.

### Ultrasonography

Ultrasonography was undertaken, utilizing GE Healthcare LOGIQ E9 ultrasonographic system with 6-15 MHz linear array transducer (GE Healthcare Japan, Tokyo, Japan), in the following 11 muscles; the tongue, and the bilateral biceps brachii (BB), 1st dorsal interosseous (FDI), Th10 paraspinalis, vastus lateralis (VL) and tibialis anterior muscles (TA) [10]. Each muscle was transversely investigated, utilizing B-mode images at the belly of the muscle. Muscles, except for paraspinalis, were examined in the supine position with limbs extended and relaxed. Paraspinalis was studied in the lateral position with the legs flexed at the knee and pulled in towards the chest, like lumbar puncture position. The skin temperature in the upper and lower limbs was > 32°C. Settings of ultrasonography were kept at the factory preset for muscle imaging in all examinations, except for the FDI muscle, with an imaging depth of 4-8 cm. The FDI muscle was examined with that of 2.5-3 cm. A width and gain were established according to each muscle, to be extensively and properly investigated. Fasciculations were observed on the live monitor at least 3 parts of each muscle for 30 seconds. The fasciculation intensity was scored from 0 to 3 according to the following criteria; 0 = no fasciculation, 1 = intermittent fasciculation in one part, 2 = intermittent fasciculation in two or more parts, 3 = sustainable fasciculation in the two or more parts.

### Statistical analysis

Data for fasciculation intensity are presented as median (inter-quartile range). To analyze side differences in fasciculation intensity and frequency, Wilcoxon signed rank test and Fisher's exact test were performed respectively. Factors which affect fasciculation intensity and frequencies were analyzed, utilizing univariate and multivariate correlation analyses. If factors fulfilled |R| > 0.2 and *p* < 0.1 in univariate analyses, those factors were subsequently included into multivariate analyses. A *p*-value < 0.05 was judged as statistically significant in these analyses. In correlation analyses, both |R| > 0.2 and *p* < 0.05 was evaluated as significant correlation. JMP Pro 13.2.0 (SAS Institute) was used in those procedures.

## Results

### Characteristics of subjects

A total of 1166 muscles of the 106 ALS patients (52 males) were investigated. Of these, data of the tongue muscle in 2 patients were excluded due to continuous voluntary movement. Their mean age was 66.9 years (range, 19 to 86 years). They fulfilled the revised El Escorial criteria, 39 with definite, 60 with probable, and 7 with possible ALS. The mean disease duration was 16.0 (SD, 16.4) months. The first symptoms affected the bulbar region in 43 patients, and the limb region in 63 (upper limb in 41). Of the 41 patients with upper limb onset, 21 patients had right side onset, 20 patients had left side onset. Similarly, of the 22 patients with lower limb onset, 10 patients had right side onset, 8 patients had left side onset, 3 patients had simultaneous onset on both sides and 1 patient was uncertain in onset side. Of the106 patients, 100 patients were right-handed, and 4 patients were left-handed. The remaining 2 patients were uncertain in handedness. Of 41 patients with upper limb onset, all patients were right-handed.

### Detection of fasciculation

**Fig. 1:**
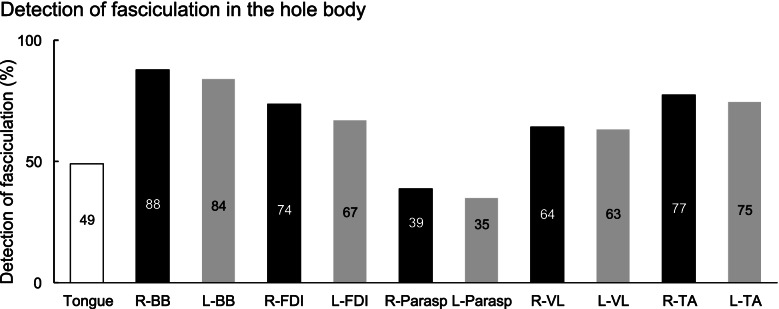
Detection of fasciculation in the hole body. Graph shows the rate of detected fasciculation. Black graph shows right side and gray shows left side. Side difference did not show in each the muscle

Fasciculations in the limb muscles were frequently found, compared with the tongue and paraspinal muscles (Fig. 1). Differences in each muscle between right and left sides were not significantly different. Those distributions were almost similar in patients with bulbar onset (tongue 48%, right BB 88%, left BB 84%, right FDI 67%, left FDI 70%, right paraspinalis 30%, left paraspinalis 30%, right VL 70%, left VL 61%, right TA 77% and left TA 77%), upper limb onset (tongue 52%, right BB 83%, left BB 88%, right FDI 76%, left FDI 68%, right paraspinalis 42%, left paraspinalis 37%, right VL 59%, left VL 66%, right TA 81% and left TA 76%) and lower limb onset (tongue 46%, right BB 96%, left BB 77%, right FDI 82%, left FDI 59%, right paraspinalis 50%, left paraspinalis 41%, right VL 64%, left VL 64%, right TA 73% and left TA 68%). Additionally, these distributions did not show significant differences between right and left sides. Handedness analyses also did not show significant differences between dominant side (FDI; 75%, BB; 87.5%) and non-dominant side (FDI; 66.3%, BB; 87.3%).

### Fasciculation intensity

**Fig. 2:**
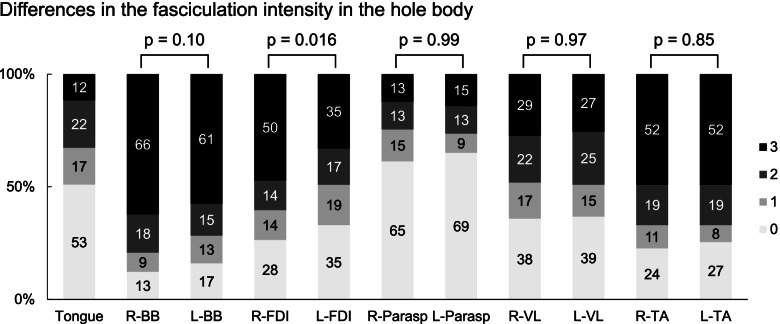
Differences in the fasciculation intensity in the hole body. Graph shows the rate of fasciculation intensity in the hole body. The fasciculation intensity in the right FDI was significantly greater than the left FDI (*p* = 0.016)

The fasciculation intensity was also more prominent in the limbs, compared with the tongue and truncal muscles (Table 1). Side difference was found in the FDI (*p* = 0.016) (Fig. 2). When patients were divided according to onset site, fasciculation intensities were almost similar among onset sites (Table 2).

**Table 1:** Fasciculation intensity in the whole body of patients with ALS

	Side		
	Right	Left	*p*-value
Fasciculation score			
Tongue	0 (0 - 2)		N/A
Biceps brachii	3 (2 - 3)	3 (1 - 3)	0.10
1st dosal interosseous	2 (0 - 3)	1.5 (0 - 3)	0.016
Paraspinalis	0 (0 - 1.25)	0 (0 - 2)	0.99
Vastus lateralis	1 (0 - 3)	1.5 (0 - 3)	0.97
Tbialis anterior	2.5 (1 - 3)	3 (0 - 3)	0.85

Fasciculation score was defined as follows; 0 = No fasciculation, 1 = Intermittent fasciculation in the one part, 2 = Intermittent fasciculation in two or more parts, 3 = Sustainable fasciculation in two or more parts

Data are given as median (IQR)

**Table 2:** Fasciculation intensity depending on onset sites

	Side		
	Right	Left	*p*-value
Fasciculation score			
Bulbar onset (*n* = 43)			
Tongue	0 (0 - 2)		N/A
Biceps brachii	3 (1 - 3)	3 (1 - 3)	0.14
1st dosal interosseous	2 (0 - 3)	2 (0 - 3)	0.78
Paraspinalis	0 (0 - 1)	0 (0 - 1)	0.77
Vastus lateralis	1 (0 - 2)	1 (0 - 2)	0.094
Tbialis anterior	2 (1 - 3)	3 (1 - 3)	0.51
U/E onset (*n* = 41)			
Tongue	1 (0 - 2)		N/A
Biceps brachii	3 (2 - 3)	3 (2 - 3)	0.27
1st dosal interosseous	3 (0.5 - 3)	1 (0 - 3)	0.022
Paraspinalis	0 (0 - 2)	0 (0 - 2)	0.77
Vastus lateralis	1 (0 - 3)	2 (0 - 3)	0.21
Tbialis anterior	2 (1 - 3)	2 (0.5 - 3)	0.69
L/E onset (*n* = 22)			
Tongue	0 (0 - 1.25)		N/A
Biceps brachii	3 (2 - 3)	2.5 (0.75 - 3)	0.018
1st dosal interosseous	2 (1 - 3)	1.5 (0 - 3)	0.11
Paraspinalis	0.5 (0 - 2.25)	0 (0 - 3)	1.00
Vastus lateralis	2 (0 - 3)	2 (0 - 3)	0.83
Tbialis anterior	2.5 (0.75 - 3)	2 (0 - 3)	0.57

Data of fasciculation score are given as median (IQR)

*U/E* Upper extremities, *L/E* Lower extremities

**Fig. 3:**
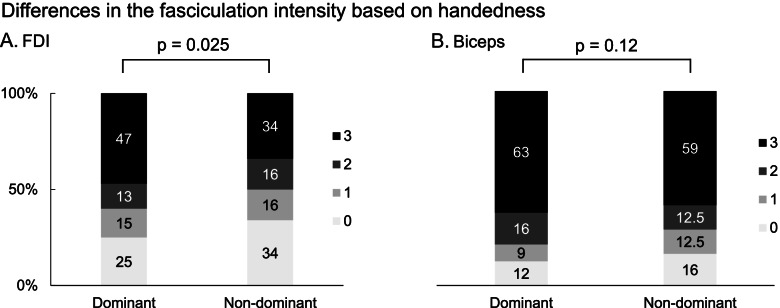
Differences in the fasciculation intensity based on handedness. Graph shows the rate of fasciculation intensity based on handedness. The fasciculation intensity in the dominant FDI was significantly greater than the non-dominant FDI (*p* = 0.025) (**A**). In contrast, fasciculation intensity in the dominant biceps was also greater than non-dominant biceps but the difference did not reach the statistically significant (**B**)

Handedness analyses disclosed that the fasciculation intensity in the dominant FDI was greater than the non-dominant FDI (dominant FDI; 2 [1 - 3], non-dominant FDI; 1.5 [0 - 3], *p* = 0.025) (Fig. 3A). In contrast, fasciculation intensities in the BB were similar in dominant and non-dominant sides (dominant BB; 3 [2 - 3], non-dominant BB; 3 [1 - 3], *p* = 0.12) (Fig. 3B). This difference in the FDI was prominent in patients with upper limb onset (dominant FDI; 3 [0.5 - 3], non-dominant FDI; 1 [0 - 3], *p* = 0.02). In contrast, the difference in the BB was not also significant in patients with upper limb onset (dominant BB; 3 [2 - 3], non-dominant BB; 3 [2 - 3], *p* = 0.27).

### Clinical factors and fasciculation intensity

Univariate analyses were undertaken to reveal clinical factors which affected fasciculation intensity in the dominant FDI and BB muscles. Gender (R^2^ = 0.048, *p* < 0.05) was related to fasciculation intensities in the FDI muscle and subsequently included into the following multivariate analysis. The multivariate analysis also showed that males (β = 0.22, *p* < 0.05) had significantly frequent fasciculations in the FDI.

Other clinical factors such as site of onset, side of onset, El Escorial criteria, age at examinations, BMI, and disease duration, were not significantly related with the fasciculation intensity.

## Discussion

Our results confirmed that fasciculations are more frequently found in the limb muscle than in the tongue and truncal muscles [17, 18], and firstly showed the intensity of fasciculations are slightly, but significantly greater for the dominant hand than in the non-dominant hand. Additionally, the fasciculation intensity in these sides was prominent in patients with upper limb onset and in male patients.

The present study showed prominent fasciculations in the dominant hand in ALS patients. A potential pathogenesis underlying handedness differences may be derived from several factors. Humans frequently use the dominant hand in daily activities, and motor neurons in the dominant hand may be exposed to prominent glutamic acid. Additionally, the dominant hand has a stronger connection to the cerebral motor cortex than the non-dominant hand [19]. In ALS, glutamate-induced excitotoxicity is one of the potential causes of motor neuron death [20], and excessive glutamate increases oxidative stress and metabolic demands and may result in motor neuron death [21]. Additionally, cortical hyperexcitability has been reported in ALS [22], and the origin of fasciculations is assumed to be such cortical hyperexcitability [5, 23]. As such, fasciculations in the dominant hand muscles may be partially derived from such excitotoxicity and metabolic disturbance. However, importantly, not only cortical hyperexcitability but also peripheral hyperexcitability are speculated to attribute to the origin of fasciculations [23]. Handedness might be a partial factor for this difference.

Fasciculation intensity in the hand muscles was prominent in male patients. Prior studies reported estradiol effects on calcium ion conductance and depress neuronal excitabilities [24–26]. Female hormone may alter axonal excitability and reduce fasciculations. Apart from it, prevalence of ALS in females is lower than males [21]. As previously described, neuronal excitability is potentially related to motor neuron death in ALS. As such, similar to differences of fasciculations, neuronal excitability differences may be associated with gender differences in prevalence.

This study has several limitations. First, examiners were blinded to the patient’s handedness, but handedness in most patients was right dominance. It might impact on results. In the future study, anonymous and stored ultrasonography images may have to be evaluated. Additionally, a recent study showed that quantitative tools for ultrasonography can detect fasciculations and would increase the robustness of the analysis [27]. Second, this study did not include many left-handed patients. In future studies, enough left-handed patients should be included. Third, this study did not consider other environmental factors. Fasciculations are affected by several factors such as drugs (including caffeine), metabolic diseases and muscle contractions [23]. This study did not examine the amount of caffeine and others. The sufficient number of patients was included in this study, but such factors might affect fasciculations.

Findings of the present study may support our clinical practice. Previous ultrasonographic studies also analyzed fasciculation frequency in ALS but did not focus on side differences [17, 18]. The present study has revealed side differences and handedness. As previously mentioned, presence of fasciculations is very important signs for ALS diagnosis. Additionally, ultrasonography is a non-invasive and convenient tool to assess fasciculations, and the combination of needle EMG with ultrasonography improves the diagnostic sensitivity [10]. As such, characteristics of the fasciculation distribution are potentially important for ALS diagnosis. Handedness and gender may have to be considered in neurophysiological testing in ALS clinical practice.

In the present study, the distribution of fasciculations in large ALS cohort could provide important information for the ALS diagnosis and suggest underlying pathogenesis. As such, systemic investigation for the fasciculation distributions in the whole body may be useful in ALS.

## Conclusion

Present study revealed that fasciculations in ALS was more prominent in the dominant hand, especially FDI. Excitotoxicity is one of the potent factors for motor neuron death in ALS. As such, frequent use of the dominant hand and male (less female hormone) may result in salient generation of fasciculations.

## Data Availability

All data generated or analysed during this study are included in this published article.

## Abbreviations

ALS: Amyotrophic lateral sclerosis
BB: Biceps brachii
EMG: Electromyography
FDI: 1st dorsal interosseous
TA: Tibialis anterior
VL: Vastus lateralis
